# Room temperature liquid metals for flexible alkali metal‐chalcogen batteries

**DOI:** 10.1002/EXP.20210182

**Published:** 2022-05-09

**Authors:** Long Ren, Bin‐Wei Zhang

**Affiliations:** ^1^ State Key Laboratory of Advanced Technology for Materials Synthesis and Processing, International School of Materials Science and Engineering Wuhan University of Technology Wuhan P. R. China; ^2^ College of Chemistry and Chemical Engineering Chongqing University Chongqing P. R. China; ^3^ Center of Advanced Energy Technology and Electrochemistry, Institute of Advanced Interdisciplinary Studies Chongqing University Chongqing P. R. China; ^4^ Institute for Superconducting and Electronic Materials Australian Institute of Innovative Materials University of Wollongong, Innovation Campus North Wollongong New South Wales Australia

**Keywords:** alkali metal‐chalcogen batteries, flexible batteries, liquid metals

## Abstract

Flexibility has become a certain trend in the development of secondary batteries to meet the requirements of wide portability and applicability. On account of their intrinsic high energy density, flexible alkali metal‐chalcogen batteries are attracting increasing interest. Although great advances have been made in promoting the electrochemical performance of metal‐S or metal‐Se batteries, explorations on flexible chalcogen‐based batteries are still limited. Extensive and rational use of soft materials for electrodes is the main bottleneck. The re‐emergence of safe liquid metals (LMs), which provide an ideal combination of metallic and fluidic properties at room temperature, offers a fascinating paradigm for constructing flexible chalcogen batteries. They may provide dendrite‐free anodes and restrain the dissolution of polysulfides and polyselenides for cathodes. From this perspective, we elaborate on the appealing features of LMs for the construction of flexible metal‐chalcogen batteries. Recent advances on LM‐based battery are discussed, covering novel liquid alkali metals as anodes and LM‐sulfur hybrids as cathodes, with the focus placed on durable high‐energy‐density output and self‐healing flexible capability. At last, perspectives are proposed on the future development of LM‐based chalcogen batteries, and the viable strategies to meet the current challenges that are obstructing more practical flexible chalcogen batteries.

## Graphical abstract

Liquid metal (LM) offers a fascinating paradigm for constructing flexible chalcogen batteries. This perspective elaborated the appealing features of LMs and discussed recent advances on LM based battery. The viable strategies and future development of LM‐based chalcogen batteries are highlighted.

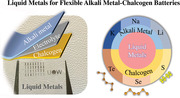

## 1 INTRODUCTION

Rigid‐type lithium‐ion batteries (LIBs) dominate the current battery technology for portable electronics due to their high energy density and long cycle‐life.^[^ ^1^ ^]^ On the other hand, new emerging higher power wearable devices^[^ ^2^ ^]^ demand the battery technology that offers, flexibility, and higher energy density.^[^ ^3^ ^]^ Recently, much effort has been put into the development of flexible batteries;^[^ ^4^ ^]^ however, improving the energy density while keeping the flexibility and lightweight of the devices is still challenging due to the lack of flexible materials as electrodes with high electrochemical capacity and energy‐density.

Taking the electrochemical potential and capacity into consideration, the electrochemistry between alkali metals and chalcogen elements (excluding oxygen) offers alkali metal‐chalcogen batteries a balanced performance toward compelling batteries.^[^ ^5^ ^]^ Therefore, flexible alkali metal‐chalcogen batteries, such as lithium‐sulfur (Li‐S) batteries, sodium‐sulfur (Na‐S) batteries, and lithium‐selenium (Li‐Se) batteries, are attractive due to the low‐cost and high theoretical capacity.^[^ ^6^ ^]^ Recently, there has been impressive progress on flexible batteries involving alkali metal‐chalcogen analogs, especially for flexible Li‐S batteries and flexible Na‐S batteries.^[^ ^7^ ^]^ Many advanced materials were developed for fabricating flexible alkali metal‐chalcogen batteries, such as the flexible cathodes consisting of sulfur hybridized with carbon nanotubes,^[^ ^8^ ^]^ graphene materials,^[^ ^9^ ^]^ carbon fibers,^[^ ^10^ ^]^ and metal‐organic framework‐based materials,^[^ ^3^, ^11^ ^]^ as well as soft anodes based on lithium metal^[^ ^12^ ^]^ and elastic quasi‐solid state electrolytes.^[^ ^13^ ^]^ Nevertheless, these materials are still not flexible enough to afford different types of deformations covering from bending, twisting, or even stretching. The insufficient performance is mainly displayed in the instability or degradation of the performance output during repeated deformation of the whole battery system. Compared to conventional batteries, the challenges toward alkali metal‐chalcogen batteries are complicated: (1) the mechanical properties of each component in the cell should be flexible, and maybe more importantly, these components should be mechanically matched and steadily connected during dynamic deformation; (2) the electrochemical activity of electrode materials should maintain a high and stable level during deforming operation, and smooth electron and ion transport through the interface are also essential; (3) the safety standards should be much stricter due to the massive mechanical strains.

**FIGURE 1:**
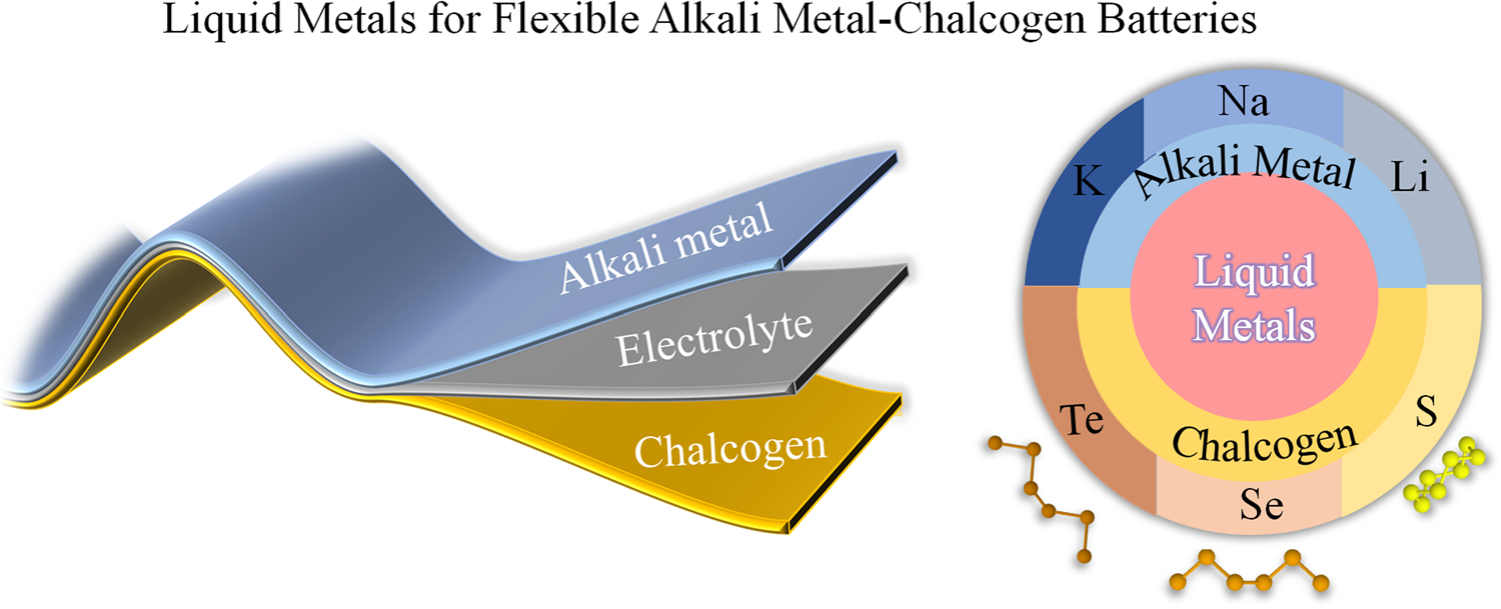
The scheme of LMs for flexible alkali metal‐chalcogen batteries

The recent emergence of liquid metals (LMs), which provide an ideal combination of metallic properties and fluidity, presents a tremendous potential for flexible electronics.^[^ ^14^ ^]^ These metals or alloys are characterized by owing low melting points which make them keep liquid state for applications. As an intrinsically flexible conductor and pool of metal ions, applying LMs in the fabrication of batteries can not only enhance the flexibility of batteries but also upgrade the operational mode of metal‐ion based secondary batteries.^[^ ^15^ ^]^ It is believed that the change transfer kinetic and the mass (ions) transport in the liquid–liquid electrode‐electrolyte interfaces would be fast for these LM‐based batteries, which results in low ohmic losses.^[^ ^16^ ^]^ Especially, the low melting point of chalcogen elements offers an intrinsic advantage to build an all‐liquid electrochemical cell. From this perspective, we focus on overviewing the current progress and then discussing the feasibility of more possibilities (Figure 1). The challenges and outlook for flexible and high‐performance LM‐based metal‐chalcogen batteries will then be considered.

## 2 LMs FOR THE ANODE OF THE CHALCOGEN BATTERY

The Li‐S battery is the most common example in the family of alkali metal‐chalcogen batteries, which has been widely investigated.^[^ ^17^ ^]^ Other than rigid‐type batteries, flexible batteries usually require more stable electrochemical activities, because they would be repeatedly bent, folded, or stretched under working conditions. Consequently, all the components of the cell, covering the current collector, anode, electrolyte, separator, and cathode, should be flexible enough to afford the mechanical deformation. Obviously, a rigid lithium metal tablet (melting point of Li ∼180°C) is not a good choice for achieving a flexible anode. This is because the dendritic growth on the surface of the anode during Li stripping‐deposition process could seriously affect the cycling performance and result in safety issues,^[^ ^18^ ^]^ which are certain to be even worse if bending or folding action is applied. Applying a metal that is liquid as anode is expected to significantly enhance the flexibility and self‐healing capability of the battery due to the infinite ductility originating from the fluidity. Furthermore, the intrinsic dendrite problem or volume expansion of alkali metals could be also eliminated by the adoption of LMs, while maintaining a durable high specific capacity.

**FIGURE 2:**
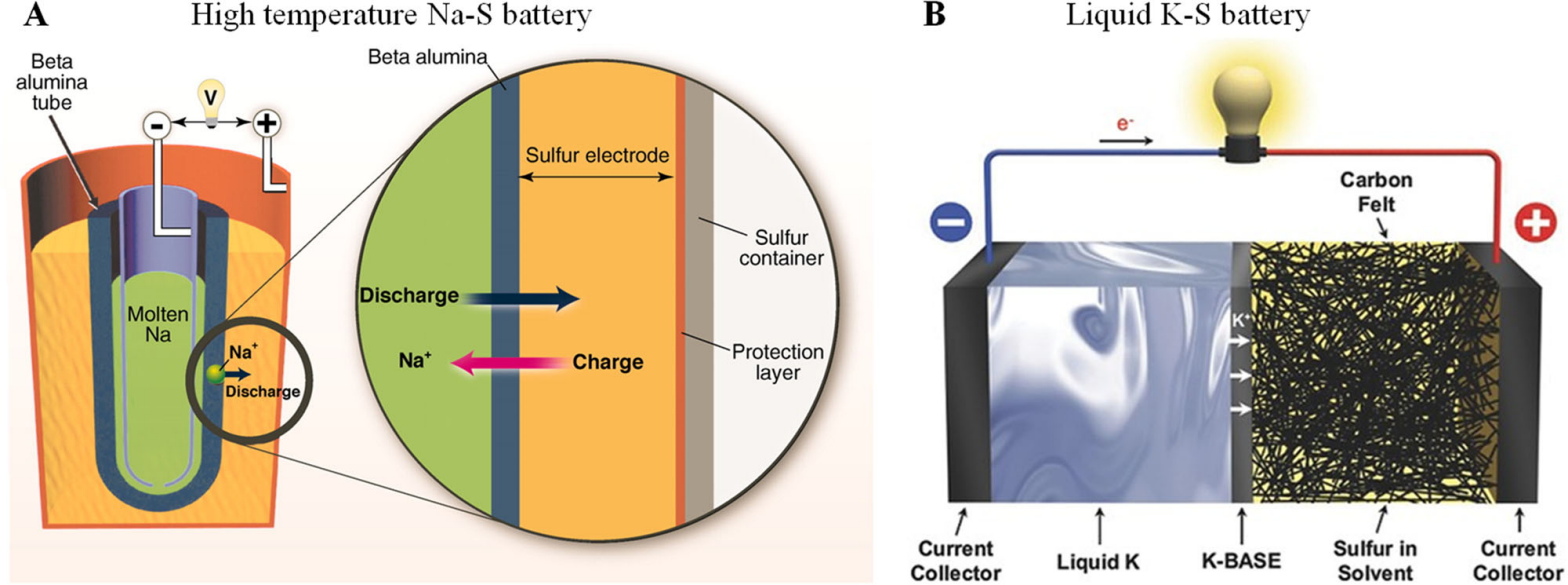
(A) Schematic representation of a high‐temperature Na‐S battery. Reproduced with permission.^[22b]^ Copyright 2011, American Association for the Advancement of Science. (B) A K‐S battery using K liquid metal and K^+^‐conducting beta‐alumina. Reproduced with permission.^[^ ^22^ ^]^ Copyright 2015, Wiley‐VCH

The invention of batteries mainly based on LM could be traced back to the 1960s, while the typical regenerative cells are composed of alkali metal negative electrodes pairing with other metallic positive electrodes.^[^ ^16^ ^]^ In the early exploration of the LM batteries, they usually operated at high temperatures >400°C, due to the fact that these LM electrodes are high‐melting‐point materials. The attractive performance of LM‐S batteries has been already demonstrated, in which the working anodes are melted alkali metals, such as Na (melting point ∼97.8°C), and K (melting point ∼63.7°C). In 1967, Kummer et al. (Ford Motor Company) invented the first high temperature (300–350°C) sodium‐sulfur (Na‐S) batteries, integrating a molten sulfur cathode, a Na^+^‐conducting β′′‐Al_2_O_3_ solid electrolyte, and a molten Na anode,^[^ ^19^ ^]^ as shown in Figure 2A. This significant exploration has aroused much interest in the field of metal/S batteries and solid‐state ionics, because of the low‐cost, high‐energy densities (760 W h kg^−1^),^[^ ^20^ ^]^ and long cycle life. Nevertheless, the high operation temperature will result in safety concerns, such as corrosion of the electrode, the fire risk of solid electrolyte, etc. The transport of the Na^+^ through the electrolyte between the S cathode and Na metal anode is displayed in Figure 2A. It can be clearly seen that during the discharge process, the Na^+^ will go through the electrolyte to arrive at the other side and react with the sulfur cathode forming various sodium polysulfide intermediates. In the charging process, reversible reactions will take place, where Na^+^ ions will be transported back to the Na metal anode. The commercial high‐temperature Na‐S batteries are predominantly used for stationary energy storage.^[^ ^21^ ^]^ Later, Lu et al. proposed a new potassium‐sulfur (K‐S) battery consisting of a liquid K anode, a sulfur cathode, and a K^+^‐conducting β′′‐Al_2_O_3_ solid electrolyte (Figure 2B).^[^ ^22^ ^]^ These K‐S batteries operated at 150°C, which is safer than the high‐temperature Na‐S batteries. Significantly, the liquid K metal presented an excellent wetting performance with the solid electrolyte compared to liquid sodium, and the battery could run for more than 1000 cycles. With the assistance of in situ Raman spectroscopy, they proposed that the mechanism during the discharge process is: K_2_S_5_ → K_2_S_4_ + K_2_S_3_ → K_2_S_3_ + K_2_S_2_. Although the K_2_S_2_ cannot be reduced to K_2_S, the K‐S cell still could achieve a capacity of 402 mA h g^−1^ even over 1000 cycles.

**FIGURE 3:**
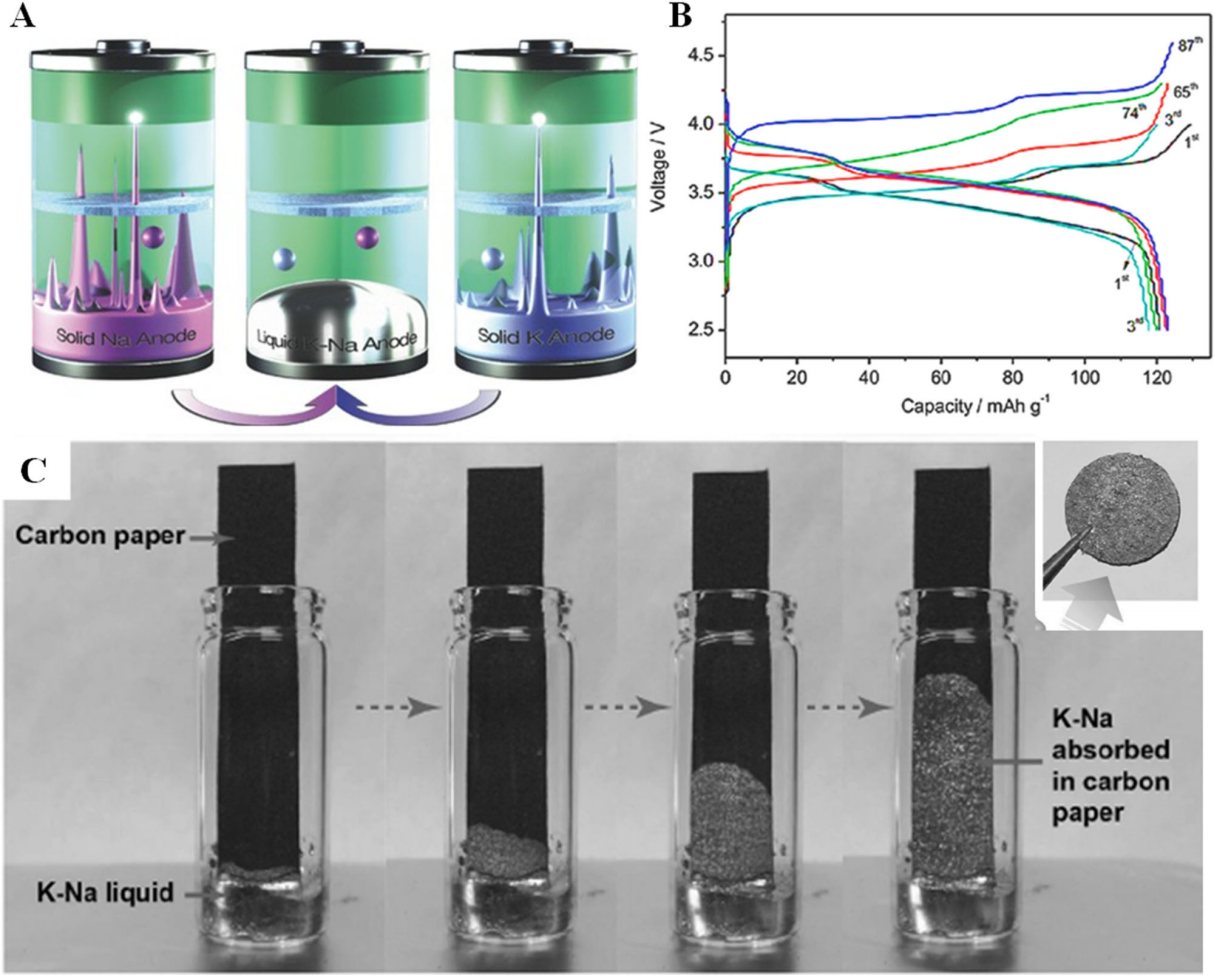
(A) Schematic illustration of dendrite‐free liquid NaK metal battery. (B) Charge/discharge curves of NaK/Na_2_MnFe(CN)_6_ cell with the initial electrolyte salt of NaClO_4_ at 1 C. (C) Photographs of liquid K–Na alloy absorbed by carbon paper. Reproduced with permission.^[^ ^23^ ^]^ Copyright 2016, Wiley‐VCH

High‐temperature LM batteries have made significant achievements, but the operation of batteries at an ambient temperature can ensure their safety and reduce the cost as well, which has attracted much attention. The alloying strategy is an effective approach to decrease the melting points of electrode materials. A good example is the NaK alloy, which is eutectic with the melting point down to −12.6°C. It could maintain the liquid state with the weight ratio of Na within the range of 9.2 to 58.2 wt% (Figure 3A).^[^ ^23^ ^]^ Moreover, this liquid NaK alloy presents good wetting with solid‐state electrolytes, which can form a stable liquid‐solid interface. The liquid NaK alloy can be fixed into a flexible porous membrane and offers a dendrite‐free anode.^[^ ^23^ ^]^ When MnFe(CN)_6_ was employed as the cathode, these LM cells operated at 25°C and delivered a high capacity of 629 mA h g^−1^ based on the Na metal as anode and 579 mA h g^−1^ for the K metal as anode (Figure 3B). This concept of LM batteries is expected to be suitable for use in flexible alkali metal‐chalcogen batteries, as the alkali metals can be absorbed in a flexible matrix and endure bending, folding, or stretching during cycling (Figure 3C).

## 3 LM HYBRIDS FOR THE CATHODE OF CHALCOGEN BATTERIES

The ultrahigh theoretical energy density of alkali metal‐chalcogen batteries originates from the chalcogen materials as cathodes. For example, Li‐S batteries possess a high theoretical capacity of 1672 mA h g^−1^, based on sulfur,^[^ ^24^ ^]^ and have a theoretical energy density of 2597 W h kg^−1^.^[^ ^25^ ^]^ In addition to its electrochemical superiority, S cathode materials display attractive advantages including nontoxicity, low cost, earth abundance, and environmental friendliness.^[^ ^26^ ^]^ Nevertheless, the shortcomings of sulfur cathodes are also obvious, such as their large volumetric expansion (∼80%), and their intrinsically insulating property, which is also true of their discharge products (for example, Li_2_S), and the shuttle effect of polysulfides.^[^ ^27^ ^]^ The poor conductivity of sulfur and the products after discharge will result in inefficient use of active materials and irreversibility in cycling, which are the major reason for their low accessible capacity.^[^ ^28^ ^]^ Additionally, the shuttle effect would also result in the loss of polysulfides into the electrolyte, and thus lead to fast capacity fading during cycling.^[^ ^29^ ^]^ These practical challenges limit the practical application of alkali metal‐chalcogen batteries.

For flexible alkali metal‐chalcogen batteries, such issues become even more serious, due to the lack of alternatives for electrode materials, especially the cathode materials. In comparison to rigid ones, the flexible chalcogen batteries have to maintain durable electrochemical performance during repeated bending or even stretching. Therefore, this requires all the electrode materials to be flexible enough to endure the possible mechanical deformation. The conventional chalcogen cathodes, such as sulfur, selenium, and tellurium, consists of active materials coated on a conductive metal foil collector. These cathodes can hardly meet the requirements of the flexible electrodes due to the possible cracking and delamination that can occur during the electrode deformation. Besides the mechanical instability, the problem of low electrical conductivity and the migration of soluble polysulfides would be intensified. Recently, a tremendous amount of work has been carried out in attempting to solve the above issues by novel chalcogen cathode hosts, including carbonaceous materials,^[^ ^30^ ^]^ polymer composite materials,^[^ ^31^ ^]^ and inorganic composite materials.^[^ ^32^ ^]^ Although these cathode hosts usually possess many merits, including high conductivity, high surface areas, and excellent flexibility, they still cannot be directly employed as single constituents of a flexible electrode.

**FIGURE 4:**
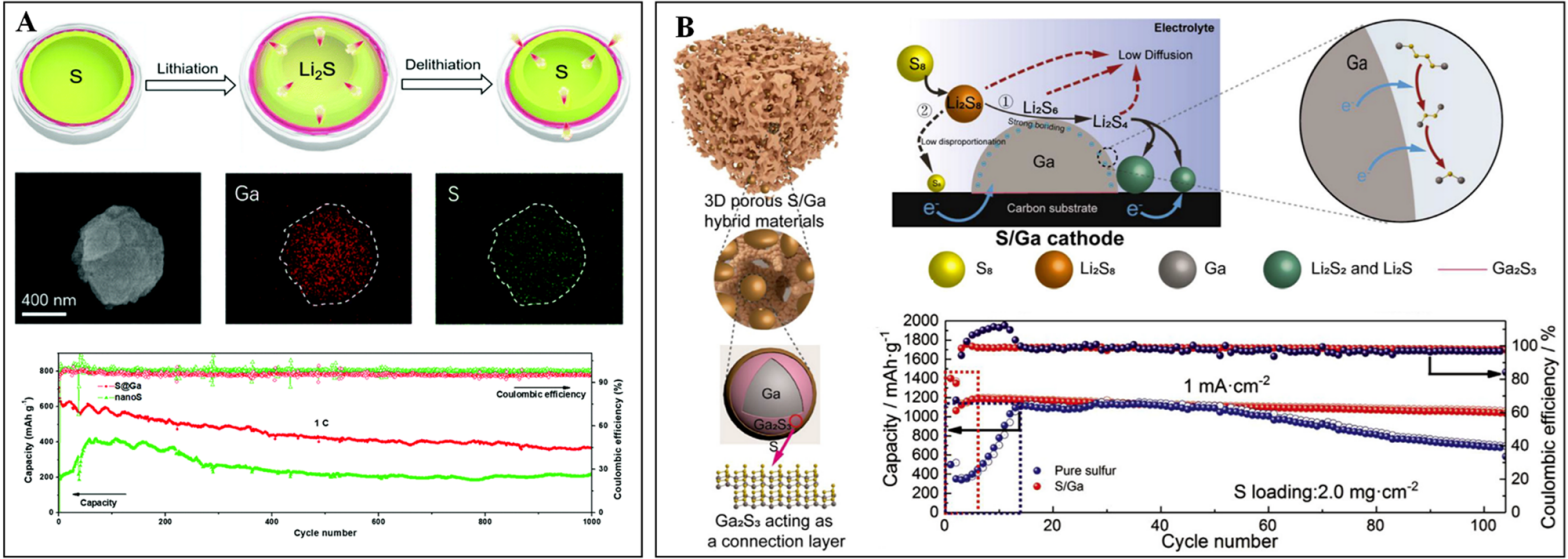
(A) Morphologies of S@Ga nanoparticles and corresponding electrochemical performance in Li‐S batteries. Reproduced with permission.^[^ ^34^ ^]^ Copyright 2019, The Royal Society of Chemistry. (B) Schematic illustration of S/Ga composite and its electrocatalytic effect toward the conversion of polysulfides for Li‐S batteries. Reproduced with permission.^[^ ^35^ ^]^ Copyright 2020, Elsevier

Due to the advantages of their metallic conductivity and fluidic compatibility, LM is an ideal mechanical and conductive agent to connect sulfur particles. Constructing hybrids by integrating LM and a chalcogen is expected to offer a long‐range interpenetrating conductive network and a mechanically robust (even self‐healing) skeleton to the cathode operation. In addition, the surface of a LM is usually electron‐rich, which can enhance the conversion of discharge product intermediates, such as polysulfides and polyselenides. For constructing a hybrid of LM and chalcogen such as sulfur, the interfacial wettability has to be considered. It is reported that, after the liquification and polymerization of sulfur, the resulted polysulfide loops/thiol groups could strongly interact with LM, which increases the interfacial strength.^[^ ^33^ ^]^ Even at the solid state, sulfur may also react with LM to form a buffer layer to enhance the wettability. For example, Zhu et al. proposed uniform S@Ga core‐shell structures as the cathode for the Li‐S batteries (Figure 4A).^[^ ^34^ ^]^ The thin Ga shells act as a wetting agent that facilitates close contact and mechanical stability. Such a Ga shell behaves like a conductive agent to improve the electron/ion transmission. More interesting, the liquid Ga shell is integrated and perfectly adapted to the volume changes during cycling, which restrains the dissolution of polysulfide and the shuttle effect. The S@Ga cathode materials displayed a high capacity of 1295 mA h g^−1^ at 0.1 C and a stable cycling performance over 1000 cycles. The adaptivity mentioned in this report can be also considered as the “self‐healing” capability of the S/Ga composite cathode since the liquid Ga can recover possible defects due to the high surface tension.

LM is not only a good conductor, but also can be regarded as a good liquid catalyst. For example, micro/nanometer scale liquid Ga materials have been considered as good catalysts for liquid‐based reactions owing to the dynamic distribution of active atoms at the surface. It is worth noting that, in comparison to solid nano‐catalyst supports, the LM‐based catalysts exhibit extraordinary longevity. In addition, liquid Ga is also regarded to favor the electrocatalytic reactions due to the high conductivity and electron‐rich surface. The electron‐rich surface of LM offers sufficient active sites to catalyze the redox reactions of lithium polysulfides, and then improve the final performance. Li et al. reported 3D porous S/Ga hybrids as cathode materials for Li‐S batteries (Figure 4B).^[^ ^35^ ^]^ The electrons tend to be transferred to the LM metal Ga and form an electron‐rich surface, which can promote the electrocatalytic conversion from lithium polysulfides to Li_2_S. Therefore, this S/Ga composite presented a specific capacity of 1044 mA h g^−1^ after 1000 cycles at the density current of 1 mA cm^−2^ with a Coulombic efficiency of 98.7%.

## 4 OUTLOOK

As we reviewed, impressive improvement and innovation have been achieved in both liquid alkali metal anodes and LM‐based chalcogen cathodes. Nevertheless, the assembly of LM‐based anodes and cathodes toward integrated flexible metal–chalcogen batteries has been not realized yet. To achieve an actual flexible battery, each cell component has to be assembled into a compact and pliable device. The current fabrication mode for LM‐based batteries is still mainly based on the standard battery components with liquid electrolyte, which is not suitable for repeatable bending or twisting. In addition, these liquid organic electrolytes may raise the possibility of electrolyte leakage and fire, raising safety risks. Using solid‐state electrolytes (SSEs) to support the electrode materials that are in the liquid state would be an ideal choice to realize the final flexibility of devices. The SSEs usually can be classified into polymer electrolytes, including solid polymer and gel polymer electrolytes, as well as inorganic electrolytes. Among these candidates, polymer electrolytes are especially preferred for flexible metal–chalcogen batteries because the inorganic electrolytes are too rigid to bear the bending or twisting.^[^ ^36^ ^]^ Recently, Liu et al. demonstrated a deformable battery based on LM materials and an elastic polyacrylic acid‐based gel electrolyte.^[^ ^37^ ^]^ With flexible electrode materials and gel electrolyte, such battery cells can be stretched from 12 to 24 cm, and show excellent shape recoverability with 98.87% performance retention during discharging. Even with these possible SSEs, the problems of interfacial contact and low ionic conductivity still need to be solved for achieving durable deformation repeatability and high charge‐discharge cycling capacity. The design concept of an integrated anode/SSE/cathode architecture is ideal for addressing these issues for the LM‐based flexible metal–chalcogen batteries, but the problems are still very challenging. Nevertheless, the development of LM‐based flexible metal–chalcogen batteries is still in its infancy, and inspiration from other more developed solid‐state batteries, for example, LIBs, will be very valuable.

Taking advantage of their fluidity, although LM anodes possess excellent mechanical pliability, challenges remain to balance the energy density, safety, and weight in practical application. Such issues would become even tougher in view of the requirements on the melting point and electrochemical reduction potential. Owning to the intrinsic strong metallic bonds, the liquid alloys that can work at room temperature are limited. Alkali alloys with low melting points, such as Na‐K alloy, are promising choices of anode materials for the direct stripping‐deposition. In view of the quite high reactivity of Na‐K alloy, even though the problem of dendrites has been overcome, the risk of the battery safety in air is still high especially in the case of packaging broken. Another thing that should be noted, the alkali alloys that are liquid at room temperature are still limited, which astricts the performance optimization and wide application. Solid‐liquid mixtures or hybrids that contain active metal and room temperature LMs may also be a possible strategy to enrich the materials variations and construct soft anodes for flexible batteries. For example, the Ga‐based liquid alloys usually have low melting temperatures and possess self‐healing capability during the electrochemical processes, but their reduction potentials are obviously higher than that of the alkali metal. Constructing Ga‐based alloys with Li or Na, or forming hybrids containing metal species and metal droplets are all optional plan. Nevertheless, the intermetallic phases formed between post‐transition metals and alkali metals can affect the final kinetic and charge transport. Establishment of electrochemical mechanism on the multi‐cation systems of these elements is highly demanded. They would be meaningful to guide the designs of LM alloy anodes and help in understanding the interfacial charge and mass transport phenomena.

To pursue high energy density, chalcogen elements should be the main active part of the cathodes. The introduction of LM could offer a flexible and conductive binder for sulfur and other chalcogens including selenium and tellurium, forming a hybrid cathode. However, the balance of the content's ratio between chalcogens‐based active materials and LMs requires careful consideration. The high content of LMs may reduce the portability and gravimetric energy density of the whole electrode. It is expected to obtain satisfied bending or self‐healing capability with the least amount of LM additives in the cathode materials. The atomic metal species in LM offer a possibility to catalytic regulate the redox kinetics of chalcogens; nevertheless, in‐depth investigations in the form of both theoretically and experimentally are highly demanded.

In conclusion, with further explorations on the design of LM favored electrolyte, LM anode, and LM‐based cathode, such novel LM‐based chalcogens battery system will promise an attractive future of applications for next‐generation flexible energy storage. It is believed that the introduction of LMs into chalcogen batteries can not only offer a design principle for high‐performance flexible batteries but also inspire extensive and in‐depth application of LMs.

## CONFLICT OF INTEREST

The authors declare no conflict of interest.
